# Preliminary Validation Study of the Intrinsic Religious Motivation Scale and the Centrality of Religiosity Scale for the Portuguese Population

**DOI:** 10.3390/ejihpe11030067

**Published:** 2021-08-18

**Authors:** Pedro Araújo, Sara Gomes, Diogo Guedes Vidal, Hélder Fernando Pedrosa e Sousa, Maria Alzira Pimenta Dinis, Ângela Leite

**Affiliations:** 1 Department of Psychology, Faculty of Philosophy and Social Sciences, Portuguese Catholic University, Rua de Camões 60, 4710-362 Braga, Portugal; s-pearaujo@ucp.pt (P.A.); s-sfcgomes@ucp.pt (S.G.); 2 UFP Energy, Environment and Health Research Unit (FP-ENAS), University Fernando Pessoa (UFP), Praça 9 de Abril 349, 4249-004 Porto, Portugal; diogoguedesvidal@hotmail.com; 3 Department of Mathematics (DM. UTAD), University of Trás-os-Montes and Alto Douro, Quinta de Prados, 5001-801 Vila Real, Portugal; hfps@utad.pt

**Keywords:** centrality of religion, Centrality of Religiosity Scale (CRS), extrinsic religious motivation, intrinsic religious motivation, Intrinsic Religious Motivation Scale (IRMS), religion, self-compassion, Self-Compassion Scale (SCS), validation study

## Abstract

Religion is a construct widely present in most people’s lives. Religious motivations, either intrinsic or extrinsic, as well as religious centrality, are crucial aspects of religion. In the Portuguese population, there are no validated instruments to assess these aspects of religion. Accordingly, this study intends to validate the Intrinsic Religious Motivation Scale (IRMS) and the Centrality of Religiosity Scale (CRS) for the Portuguese population. This is a validation study whose sample consists of 326 participants (73.1% women) from the general population. Exploratory and confirmatory factor analyses were carried out and the correlations between the constructs and self-compassion were determined to assess the convergent and divergent validity. The results obtained confirm the existence of models adjusted to the population, allowing us to conclude that the instruments are reliable for assessing the studied constructs. The validation of the IRMS and CRS for the Portuguese population is of outstanding importance, as it provides researchers in the field with valid instruments and psychometric qualities to carry out research within religion and religiosity.

## 1. Introduction

### 1.1. Religion and Religiosity

Religion is defined as the whole set of symbols and meanings, with a range of its own beliefs and rituals, shared by a whole set of people [1,2]. It usually focuses its attention on what is supernatural, sacred, or divine and in all phenomena that occur outside of what are considered to be the natural laws [3]. It can be considered as a constituent part of a broader social or cultural context [4], being, therefore, a form of culture whose objective is centred on the attribution of meaning, when it is necessary, and also on a structure suitable for beliefs, expectations, and actions [5]. Religion is, therefore, an important element to consider in people’s lives [6]; by resorting, for example, to religious coping strategies. The general research realized that these allow greater spiritual growth, positive affection, and self-esteem. It is also observed that individuals who resort to these religious strategies demonstrate less depression, anxiety, and distress, among others [7].

Often, the religiosity term is used instead of religion, so it is important to make a distinction between these two constructs. Religiosity is the degree to which a person is religious, regardless of their specific religious beliefs or the way they manifest themselves [8]. Religiosity refers to the type of subjective response that the individual elaborates when searching for existential meaning, which is also underlying a transcendent entity [9]. Thus, religiosity is the subjective component of religion, exposing the idiosyncratic psychological and human reality of the individual in the search for meaning and having in the answer, a dimensional base that transcends the individual [9]. Within the sphere of religiosity, one can distinguish the concept of religious identity, i.e., the importance of belonging to a religious group, and religious practice, i.e., the involvement of group members in their devotion and in following religious laws, identifying themselves with the same religious practice [10,11].

### 1.2. Psychology of Religion

“The psychology of religion consists of the systematic application of psychology’s methods and interpretive frameworks to the broad domain of religion” [12] (p. 1). Some psychologists have characterized religious beliefs as pathological [13]. According to Freud, the concept of God is essentially the product of an unconscious anthropomorphic construct. Researchers who study the psychology and neuroscience of religion found that religion may be a by-product of the way our brains work. Additionally, people also have a bias for believing in the supernatural [14]. The cognitive science of religion explores causal explanations of religious influence (thoughts, ideas, practices, and experiences) [15] and Kelemen and Rosset [16] (p. 1) showed that, in situations of high cognitive demand, even science-educated adults present signs of “scientifically unwarranted teleological explanations as correct”.

The psychology of religion is not just dedicated to studying religious identity, religious practice, and beliefs. It is also dedicated to studying non-believers and non-religion [2,17]. In fact, non-belief development is based on psychological mechanisms that support sceptical thinking and critical reasoning, reducing the plausibility of religious ideas [17]. According to Anczykand and Grzymała-Moszczyńska [18], contemporary psychology of religion faces many challenges, namely, cross-disciplinary interconnections, responses to societal challenges, and research methodology. This last challenge includes the development and validation of instruments to better study the phenomenon of religiosity from a psychological perspective.

### 1.3. Religious Motivation

Depending on the type of motivation towards religion that a subject incorporates, two types of orientation can be found, intrinsic and extrinsic. An intrinsic orientation refers to taking religion as a motivation for life [19], and someone who has this type of orientation tends to be motivated by spiritual goals [20,21]. Someone oriented towards intrinsic religiosity may be motivated by issues related to religious identity because a person with this orientation acts as if they lived the religion [22,23]. On the opposite pole, an extrinsic orientation is defined as one in which religion is used in order to achieve a state of well-being [19], thus fulfilling some socialization or status needs [20]. Thus, a type of extrinsic orientation uses religion as a way to obtain approval and support, focusing only on the behavioural and social aspects of religion [8]. Briefly, the one who is intrinsically motivated lives religion, and the one who is extrinsically motivated uses religion [20].

One of the instruments that is most frequently used in order to measure religiosity is based on Gordon Allport’s [20,24] proposal to attribute these two dimensions to this construct: intrinsic and extrinsic [8]. With relation to the measurement of religiosity itself, some studies show that female individuals tend to exhibit a higher level of religiosity than men [25,26] and a lower level of extrinsic religiosity [22,27]. In contrast, some authors did not find any relationship between religion and gender [28], and others did not find significant gender differences in intrinsic religiosity [22] although it should be noted that, in the latter case, the sample used mainly included males.

Batson [29] considered that Allport [20] should have included facets of mature religion in his measurement of intrinsic religiosity. To fill this gap, Batson et al. [30,31] suggested a new religious motivation called “Quest.” Quest assesses religious orientation that “involves honestly facing existential questions in all their complexity, while at the same time resisting clear-cut, pat answers” [30] (p. 166). In sum, Quest is a form of religiosity that views questions and their answers as of equal importance [30,31].

### 1.4. Centrality of Religiosity

Huber [32] introduced the concept of the centrality of religiosity, affirming the importance of religious constructs among other constructs in someone’s personality. The centrality of religiosity is related to the effectiveness of religion. The more central the religion, the greater its impact on a person’s experience and behaviour [33]. A similar idea was described by Park [34], who stated that religious meaning systems are the lens through which experiences are interpreted and assessed. Huber [32] was inspired by Glock [35], whose approach has its origins in the sociology of religion, from the perspective of personality psychology, particularly influenced by the concepts of intrinsic and extrinsic religiosity authored by Allport and Ross [20] and by the concepts of psychology of the personal religious construction system of Neff [36]. Religiosity is associated with resilience, a personality characteristic that reflects one’s ability to effectively adjust and adapt to challenging the circumstances of life [37]. This association is strengthened by hope, as a mediator, and by positive affect (but not negative affect), as a moderator in the indirect effect of the religious meaning system to resilience through hope [37]. Stressful life events are often linked to psychological symptoms through their influence on meaning systems, i.e., an individual’s views about the world, namely, religious meaning systems [38]. Besides, meaning violations and religious and or spiritual struggles arise, responding to stressful life events and are associated with Post Traumatic Stress symptoms [38].

Glock [35] defined five central dimensions of religiosity that constitute a general frame of reference for empirical research: the intellectual, ideological, ritualistic, experiential, and consequential dimensions. Stark and Glock [39] eliminated the consequential dimension from the religiosity model and divided the ritual dimension into public and private practice, thus maintaining five dimensions. Glock’s [35] approach was essentially directed towards religious institutions and social expectations [33].

### 1.5. Self-Compassion

Positive psychology is an area focused on enhancing a subject’s strengths and values, not focusing on pathologies [40]. Thus, by enhancing, for example, high levels of mental well-being, positive psychology is able to prevent the appearance of problems related to mental health [41]. A construct of positive psychology that has been the focus of attention more recently is self-compassion as it demonstrates to be related to good mental health [42]. By self-compassion, the subject’s ability to be kind and understanding with themselves and with their own weaknesses is understood, thus improving their mental suffering [43]. This construct implies that the subject has the ability to recognize that the pain and failure experienced are common to human experience and, therefore, not avoidable. It is also necessary to understand that self-compassion makes the subject aware of emotions, thus having the ability to face, without exaggeration or self-pity, certain thoughts and feelings that may be painful [44]. Finally, considering the expression of this construct through gender, Yarnell et al. [45] found that male individuals tend to have a level of self-compassion slightly higher than females, a small, but yet significant difference. As mentioned above, being a construct of positive psychology, self-compassion can promote higher levels of mental well-being, so there must be a focus on further enhancing this construct in the lives of individuals and more specifically in the female population, when considering the results of general research.

### 1.6. Religion and Self-Compassion

With regard to religion and self-compassion, there are not many current studies that aim to understand the relationship between the two variables. Nevertheless, studies such as those by Ghorbani et al. [46] found that self-compassion is positively correlated with attitudes towards religion and also with religious orientations, both intrinsic and extrinsic. In addition to this, other studies showed that self-compassion predicts stronger religious commitment and consequently better mental health [47]. Some studies also report that certain schools of religion emphasize self-compassion as a value, pointing out that, when associated with religious orientations, it can improve the individual’s mental health [48,49]. This is evidenced as these schools claim that God expresses compassion towards human beings and, therefore, we should not blame ourselves for the problems that overtake us [2]. Based on this, authors such as Rezapour-Mirsaleh et al. [48] seek to prove that an intervention in self-compassion based on religion can be effective for individuals who, in their lives, may have some kind of unresolved problem, preventing the emergence of problems related to mental health, such as mentioned by Forsman et al. [41] about positive psychology.

### 1.7. Objective and Hypotheses

The general objective of this study is to validate the Intrinsic Religious Motivation Scale (IRMS) (10-item version) and the Centrality of Religiosity Scale (CRS) (5-item version) for the Portuguese population, assuming that a good adjustment model will be found for each of these scales (***H1***) and that these scales would present a good convergent and divergent validity (***H2***).

## 2. Materials and Methods

This is a cross-sectional validation study, of a quantitative nature, the collection of data on the issues under study being carried out in a single moment.

### 2.1. Procedure

All procedures carried out in this study are in accordance with the Declaration of Helsinki [50], and this study was approved by the Ethics Committee of the Regional centre of Braga, Universidade Católica Portuguesa in 2020 January (date acting as reference ID). Authorization was requested, and granted, from the authors of the original scales. The scales were translated and back-translated by researchers, a theologian, and two bilingual psychologists. First, they were freely translated into Portuguese by two researchers separately. Then, the two versions were compared and a consensus was reached. Finally, this version was translated into English by a third investigator and compared with the original version. Data collection took place through the Google Forms online platform and the questionnaire was disseminated via email and through social networks. The questionnaire includes informed consent in which the main objective of the investigation is defined and the confidentiality and anonymity of the data are guaranteed. The participant must give his consent in order to proceed with the participation.

### 2.2. Instruments

#### 2.2.1. Socio-Demographic Questionnaire

In order to characterize the sample of this study, socio-demographic information was collected including age, gender (0—male; 1—female), number of years of education, marital status (0—no affective relationship; 1—with an emotional relationship) and occupation (0—inactive; 1—active).

#### 2.2.2. Intrinsic Religious Motivation Scale (IRMS)

The Intrinsic Religious Motivation Scale (IRMS) was developed by Hoge [28] in order to address the lack of properly validated measurement scales of religiosity, having been conceived in line with the intrinsic religiosity conceptualized by Allport [51]. At the time of its design, a preliminary validation questionnaire included 30 items, plus eight from the study by Allport and Ross [20]. The remaining 22, out of 38, were designed from scratch. After correlations with 21 items from the Feagin [52] scale and nine items out of the 22 previously introduced, a final version with 30 items was obtained. Finally, items that intending to know the frequency of going to church and religious reading, not previously considered, were included. The long version of this scale consists of 30 items and has two dimensions, i.e., intrinsic religiosity and extrinsic religiosity. Regarding the response modalities, for the first 28 items, they appear on a Likert scale with 4 responses: completely agree, agree, disagree, and completely disagree, with a score of 1, 2, 4, and 5, respectively, for each response. Items 29 and 30 have four possible answers each, which are scored from 1 to 4. It should be noted that all items that assess the dimension of extrinsic religiosity, i.e., items 1, 4, 9, 10, 15 to 17, 20, 21, 23 to 25, 27, and 28) are inverted. With regard to the scale results, the total sum of the items must be averaged, interpreting that a higher score represents someone with extrinsic religiosity and vice versa. In turn, the short version of the scale is composed of only 10 of the 30 items, i.e., 3, 5, 6, 11 to 14, 21, 23, and 24, maintaining the response and assessment styles. Both versions of the scale have a reliability of 0.90.

#### 2.2.3. Centrality of Religiosity Scale (CRS)

The Centrality of Religiosity Scale (CRS) [32] is a measure of the centrality and importance of religious meanings in individuality. It questions the general intensity of the dimensions of religiosity, extracting from it a measure of the centrality of religiosity. CRS is based on Charles Glock’s [35] multidimensional model of religion from the sociology of religion. Huber [32] introduced the concept of the centrality of religiosity, affirming the importance of religious constructs among other constructs in someone’s personality. The centrality of religiosity is related to the effectiveness of religion. The more central the religion, the greater its impact on a person’s experience and behaviour [33]. Huber [32] elaborated a proposal of five key dimensions of religiosity, considering the ideals of Allport and Ross [20] and improving the dimensions listed by Glock [35]. This scale has been applied in more than 100 studies of sociology and psychology of religion and religious studies in 25 countries with a total of more than 100,000 participants [33].

There are six versions of the CRS (CRS-5; CRSi-7; CRS-10; CRSi-14; CRS-15; and CRSi-20) [33]. CRS has several versions in order to broaden its field of applicability, having been originally developed to measure religiosity in a monotheistic context of God, i.e., Judaism, Christianity, and Islam. In addition, there are other religions, for example in the East and other new religions in the West, which are based on meditation and another divine concept. In order to generalize these concepts, some changes were made, such as changing the expression “God” to “God or something divine” [33].

CRS-5 [33] is the less extensive CRS version, consisting of 5 items, measuring the different dimensions mentioned before, i.e., intellect, ideology, public practice, private practice, and religious experience. The response modalities for items 1 and 2 range from 1 to 5, i.e., never too often. Response modalities for item 3 range from 1 to 5, i.e., not at all too much. The response modality for item 4 varies from 1 to 6, i.e., never to more than once a week. The response modalities for item 5 range from 1 to 8, i.e., never to several times a day. The scale score results from the average value of the answers, giving rise to three distinct groups: very religious individuals (scoring between 4 and 5 values), religious individuals (scoring between 2.1 and 3.9 values), and non-religious individuals (scoring between 1 and 2 values). CRS-5 scale achieved good levels of internal consistency, with a Cronbach’s alpha (α) coefficient of 0.85, according to Huber and Huber [33].

#### 2.2.4. Self-Compassion Scale (SCS)

According to Neff [36], compassion is understood as the desire to alleviate the other’s anguish. On the other hand, self-compassion refers to the same need, but focused on the self, resorting to feelings of care and kindness in the face of the suffering that the self may experience. The Self-compassion Scale (SCS), which will be used as a measurement instrument for compassion, was originally developed and validated by Neff [36]. In SCS, the author embraced three main components in his definition of self-compassion, i.e., self-kindness, common humanity, and mindfulness. This instrument originally has 26 items that assess three dimensions: self-kindness (*α* = 0.78) vs. self-judgment (*α* = 0.77); common humanity (*α* = 0.80) vs. isolation (*α* = 0.79) and mindfulness (*α* = 0.75) vs. identification (*α* = 0.81). A five-point Likert-type response scale is presented for each item, ranging from 1, almost never, to 5, almost always. In general, the SCS demonstrated good internal consistency (*α* = 0.92). It is noteworthy the existence of a less extensive version, authored by Raes et al. [53], containing 12 items, two for each dimension—this was not used because the long version was used for the Portuguese population in the adaptation [54]. In this Portuguese adaptation, the same dimensions and the same number of items were maintained, as well as the response modality of the long scale, with a very good level of general internal consistency (*α* = 0.92).

### 2.3. Participants

The sample consists of 326 participants and the presence of a greater number of female subjects is highlighted, as well as a large majority of active subjects. The mean age is about 31 years old, meaning that most of the sample is in a romantic relationship (Table 1).

### 2.4. Statistical Analysis

The data referring to the sample of this study were treated using the statistical analysis software Statistical Package for Social Sciences (SPSS, version 27) and AMOS, also version 27. The analyses carried out in this study were descriptive analyses (mean, deviation- standard, minimum and maximum, asymmetry and kurtosis), Cronbach’s alpha reliability analyses, and correlation analyses (Pearson’s r and Spearman’s rho). To proceed with the validation of the IRMS and the CRS, multivariate techniques were used. More specifically a factor analysis was used, firstly carrying out an exploratory factor analysis (EFA) in order to reduce and identify the variables, grouping them into factors that may be more representative and, finally, a confirmatory factor analysis (CFA) was carried out in order to confirm whether the model found in the previous analysis presents good quality.

EFA (maximum likelihood) with principal component analysis (PCA) was carried out for the 21 motivation items by running an orthogonal Varimax rotated analysis to obtain a factor structure for the variables in the study. Sample adequacy was assessed using Kaiser-Meyer-Olkin (KMO) value [55] and Bartlett’s Test of Sphericity [56]. Factors were assessed using Eigenvalues higher than 1 [57] and a minimum of 3 items per factor [58]. Items were removed based on commonalities (<0.30), factor loadings (<0.40), matrix correlation (<0.30), and if Cronbach’s alpha increased, the item is deleted.

CFA with a robust maximum likelihood estimation was conducted with the Satorra and Bentler [59] corrected chi-square values (*χ*2 < 2) being applied using AMOS 27. Comparative fit index (CFI), Tucker–Lewis index (TLI), and the root mean square error of approximation (RMSEA) were used to assess overall global model fit. Higher values for CFI and TLI and lower values for RMSEA indicated a better fit. CFI and TLI ≥ 0.90 and RMSEA ≤ 0.08 were criteria for adequate model fit, whereas CFI and TLI ≥ 0.95 and RMSEA ≤ 0.06 were criteria for well-fitting models [60]. Browne and Cudeck [61] used the definition of “close fit”, being that PCLOSE gives a test of close fit (≥0.05). Standardized Root Mean Square (SRMR) allows assessing the average magnitude of the discrepancies between observed and expected correlations as an absolute measure of (model) fit criterion, and it should present <0.08 value [62].

Convergent validity was calculated by composite reliability (CR) and average variance extracted (AVE) values. Discriminant validity was assessed by the square roots of the AVE values and its comparison with IRMS and SCS, and CRS and SCS dimensions cross-correlations (Pearson correlation). CR should be from 0.60 and above, depending upon how many items the scale has. Smaller numbers of scale items tend to result in lower reliability levels, while larger numbers of scale items tend to have higher levels. The value of AVE for each construct should be at least 0.50 [63]. The square root of AVE should be larger than the surrounding numbers in the correlation table. Significance was set at *p*  <  0.05.

## 3. Results

### 3.1. Validation of Intrinsic Religious Motivation Scale (10 Items)

#### 3.1.1. Exploratory Factor Analysis (EFA)

Considering the ten-item version, an EFA was performed (Table 2) in which the items were distributed by two factors; items that constitute factor 1 refer to intrinsic motivation, and those that contribute factor 2 to extrinsic motivation, explaining 67.08% of the total variance, showing good psychometric qualities.

#### 3.1.2. Confirmatory Factor Analysis (CFA)

**Figure 1:**
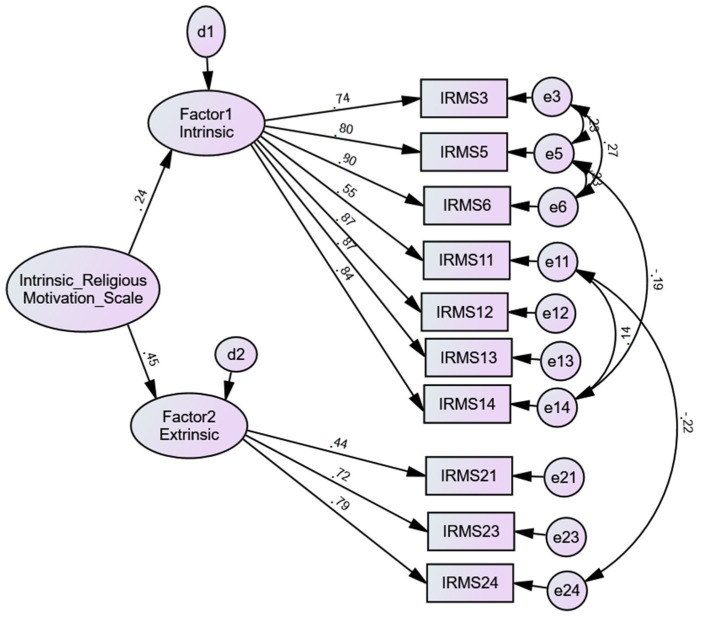
Confirmatory Factor Analysis of the Intrinsic Religious Motivation Scale (IRMS) (10 items), (*n* = 326).

AFC was carried out based on this last ten-item model. It was noticed that without modification indices the value of the model was not good [*χ*2(34) = 4.14; *p* < 0.000; CFI = 0.94; TLI = 0.92; RMSEA = 0.10; PCLOSE = 0.000; SRMR = 0.058]. Therefore, the modification index was used and established the correlations between the errors, as shown in Figure 1, in which it was found that IRMS had a good fit model [*χ*2(28) = 2.56; *p* < 0.000; CFI = 0.98; TLI = 0.96; RMSEA = 0.07; PCLOSE = 0.05; SRMR = 0.05], although the modification indices have suggested correlations between some errors, which were accepted because they occur within the same factor, except for the correlation between the error in item 11 and the error in item 24 (Figure 1).

Table 3 shows the descriptive analysis of the total and respective factors of the 10-item version of the IRMS according to the model found after EFA and CFA. Based on this, it is possible to verify satisfactory levels of reliability, the only lowest being the extrinsic factor. In this way, it was possible to replicate the structure proposed by the authors and achieve a proper separation of the two types of items, i.e., intrinsic and extrinsic.

### 3.2. Validation of Centrality of Religiosity Scale (5 Items)

#### 3.2.1. Exploratory Factor Analysis (EFA)

In the EFA of the CRS (Table 4), a structure was found in which the 5 items were grouped into a dimension, explaining 70.73% of the total variance.

#### 3.2.2. Confirmatory Factor Analysis (CFA)

**Figure 2:**
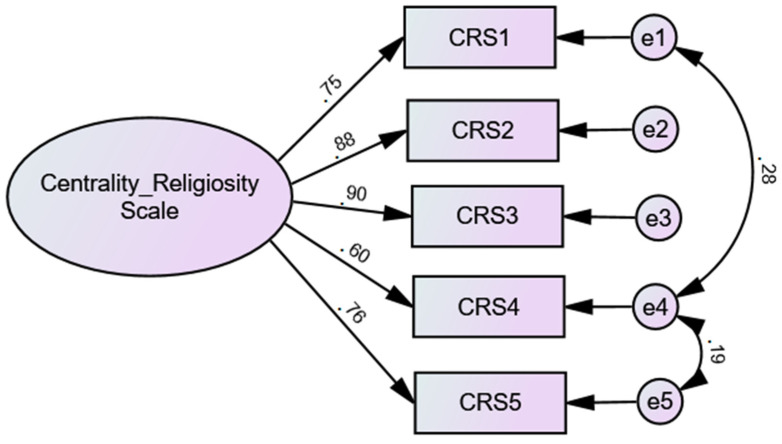
Confirmatory Factor Analysis of the Centrality Religiosity Scale (CRS) (5 items) (*n* = 326).

CFA of the 5 items for the Portuguese sample was carried out, with the objective of confirming the model found in the EFA. It was found that the CFA of the CRS presented an excellent adjustment model [*χ*2 (3) = 0.94; *p* < 0.42; CFI = 1; TLI = 1; RMSEA = 0.00; PCLOSE = 0.71; SRMR = 0.09], although the modification indices suggested correlations between some errors, which was accepted as they all occur within the same factor (Figure 2). It was possible to replicate the structure proposed by the authors.

Concerning the descriptive analysis of the total of the 5-item version of the CRS according to the model found after EFA and CFA, M = 3.24, SD = 0.89, MIN = 1, and Max = 5 were found. Regarding reliability, Cronbach’s alpha values in the original version were 0.85 and 0.89 in this study.

### 3.3. Association between Intrinsic Religious Motivation Scale and the Centrality of Religiosity Scale and Sociodemographic Variables

Correlations were established between the two scales and sociodemographic variables. In these correlations, age is negatively and significantly correlated with the total IRMS (*r* = −0.20; *p* < 0.001) and with the intrinsic factor (*r* = −0.17; *p* < 0.001). However, the value of correlations is low. Marital status is negatively and significantly correlated with the total IRMS (*rs* = −0.12; *p* > 0.05), with the intrinsic factor (*r* = −0.14; *p* > 0.05). Since the correlation between the 10-item version (total and subscales) correlated with marital status, a Student’s *t*-test for independent samples was carried out. It was found that there are statistically significant differences between marital states regarding the full scale [*t*(198.40) = −2.42; *p* = 0.02; *d* = −0.30] and the intrinsic factor [*t*(324) = −2.50; *p* = 0.01; *d* = −0.29]. People who are in an emotional relationship score higher on both dimensions than those who are not.

Age is positively correlated with the centrality of religion (*r* = 0.24, *p* < 0.001). There are no statistically significant differences in the averages of centrality of religion as a function of gender. Marital status is significantly negatively correlated with the centrality of religiosity (*r* = −0.118; *p* < 0.05). Student’s *t*-test was carried out considering the understanding of the described relationship and it was found that people who are not in an affective relationship (*M* = 3.09, *SD* = 1.17) have significantly higher values [*t*(324) = 2146; *p* = 0.033; *d* = 0.25] than those in an affective relationship (*M* = 2.81, *SD* = 1.10).

### 3.4. Association between Intrinsic Religious Motivation Scale and Self-Compassion Scale

Table 5 shows the descriptive statistics of the total SCS and respective dimensions (Table 5) and its reliability. It is possible to verify that all dimensions, as well as the total SCS, present an adequate internal consistency, with values above 0.70 [64]. In turn, Cronbach’s alpha values are close to the values of the original study [36] and higher than the values obtained in the validation study of the scale for the Portuguese population [54]. Furthermore, it is possible to see that the dimensions with the highest averages are common humanity and mindfulness.

Regarding the correlations between IRMS and SCS, there are positive and significant correlations between the extrinsic factor and the common humanity dimension (*r* = 0.19; *p* < 0.001) and mindfulness (*r* = −0.16; *p* < 0.05). There is also a negative and significant correlation between extrinsic factor and isolation (*r* = −0.12; *p* < 0.05) (Table 6). CR, AVE, and the square root of AVE are above the recommended values. Thus, both convergent validity and discriminant validity were statistically verified.

### 3.5. Association between the Centrality of Religiosity Scale and Self-Compassion Scale

H2 predicted that a higher religiosity would be positively correlated with a higher self-compassion. This hypothesis was partially confirmed as there are only significant correlations between the CRS and the Total SCS and between the CRS and the SCS isolation subscale (Table 7). CR, AVE, and the square root of AVE are above the recommended values. Thus, both convergent validity and discriminant validity were statistically verified.

## 4. Discussion

The general objective of this study is to validate the IRMS (10-item version) and the CRS (5-item version) for the Portuguese population, assuming that a good adjustment model would be found for each of these scales (***H1***) and that these scales would present a good convergent and divergent validity (***H2***).

Regarding the ***H1***—i.e., the expectation to find a good adjustment model for the IRMS and CRS for the Portuguese population—it was possible to find a good adjustment model for the 10-item version of the IRMS and for the 5-item version of the CRS for the Portuguese population. In fact, in both cases, the model found replicates the model of the original authors (10 items and two factors for IRMS and five items and 1 factor for CRS) and the psychometric qualities of the two scales are very good (assessed by Cronbach’s alpha, CR, AVE and correlations). These results are in line with those found in other populations [20,22,25]. With regard to IRMS, it was at the base of the construction and validation of the Arabic Scale of Intrinsic Religiosity [21]. Hafizi et al. [23] validated the IRMS for Farsi (in a population of Muslims), and this version presented good psychometric quality. Regarding CRS, Esperandio et al. [24] also found a good validation adjustment model for the Brazilian population, having revealed good psychometric qualities in the Brazilian cultural context. Regarding the validation of the CRS for the population of Georgia, United States, it presents acceptable and conventional indices, allowing the interpretation of individual parameters [27].

The second hypothesis, ***H2***, i.e., IRMS and CRS would present a good convergent and divergent validity was also confirmed. In fact, both the IRMS and the CRS presented AVE and CR values above those recommended, as well as presenting significant correlations with the SCS, although not very expressively in regard to the IRMS. Furthermore, squared root AVE values were above the correlations found. Thus, the convergent and divergent validity of the two scales was demonstrated. These results are consistent with those that the authors of other versions of these instruments have found [21,23,24,27].

Correlations were found between the extrinsic factor of intrinsic religious motivation and dimensions of the SCS, i.e., positive correlations were found with the dimensions of common humanity and mindfulness and negative ones with isolation. These correlations contradict that greater intrinsic religiosity potentiates greater self-compassion. However, the correlations were found to corroborate the results found by Ghorbani et al. [46], who state that self-compassion is positively correlated with both intrinsic and extrinsic orientations. Significant correlations were also found between CRS and total SCS and between CRS and the isolation subscale [46,47,48]. This is in line with the study by Souza et al. [49], who found a significantly positive correlation between frequency of religious practice and self-compassion.

## 5. Conclusions

The aim of this study was to validate instruments for the Portuguese population to assess religious motivation, as well as the centrality of religiosity, and this objective was achieved. The validation of the IRMS and CRS for the Portuguese population is of outstanding importance, as it provides researchers in the field with valid instruments and psychometric qualities to carry out research in religion and religiosity.

With regard to limitations, the sampling method used is noteworthy, since the context of isolation and social isolation that occurred during the data collection, related to the COVID-19 pandemic lockdowns, restricted the availability of questionnaires, to be published exclusively online. Another factor that may have prevented more accurate results is the fact that self-report instruments were used exclusively. The exclusive use of these instruments prevents the objectivity of the answers, thus the answer given by the participants may not correspond in the best way to their ideas.

Future studies should use larger samples and, ideally, qualitative investigations should be designed in order to better understand the participants’ religious motivation.

## Figures and Tables

**Table 1:** Sample description (*n* = 326).

Variables	*M*	*SD*	*Min*	*Max*
Age (years)	31.49	13.56	17	73
Education (years)	14.80	4.61	1	55
Gender	***N***	***%***		
Male	88	26.9		
Female	238	73.1		
Marital status				
No effective relationship	114	35		
In an effective relationship	212	65		
Occupation				
Inactive	23	7.1		
Active	326	92.9		

Note: *M =* Mean; *SD* = Standard deviation; *Min* = Minimum; *Max* = Maximum.

**Table 2:** Exploratory Factor Analysis of the Intrinsic Religious Motivation Scale (IRMS) (10 items), (*n* = 326).

Items	*h^2^*	*L1*	*L2*
3. My faith involves all my life.	0.68	0.82	−0.07
5. One should seek God’s guidance when making every important decision.	0.74	0.86	−0.01
6. I experience the presence of the Divine in my life.	0.75	0.87	−0.01
11. My faith sometimes restricts my actions.	0.41	0.63	−0.14
12. Nothing is as important to me as serving God as best as I know how.	0.78	0.87	0.17
13. I try hard to carry my religion over into all my other dealings in life.	0.77	0.87	0.10
14. My religious beliefs are what really lie behind my whole approach to life.	0.73	0.84	0.16
21. It doesn’t matter so much what I believe as long as I lead a moral life.	0.44	0.08	0.66
23. Although I am a religious person, I refuse to let religious considerations influence my everyday affairs.	0.70	−0.08	0.83
24. Although I believe in my religion, I feel there are many more important things in life.	0.70	0.05	0.84
Eigenvalues		4.80	1.90
Total explained variance (%)		48.08	19.00
Correlational matrix range [0.30–0.90]	0.325–0.764 except items 21, 23, 24 (correlated with each other)		
Determining value [above 0.00001]	0.003		
Bartlett’s Test of Sphericity (*df*); *p* < 0.05	1845.34 (45); <0.00		
Kaiser-Meyer-Olkin (KMO) measure (above 0.50)	0.88		
Diagonal element of the anti-correlational matrix (above 0.50)	0.58–0.94		
Cronbach’s alpha (*α*)	0.84, if items 21, 23, 24 are deleted then 0.85, 0.86, 0.85		

*Notes: h^2^* = Extracted communality coefficients; *LD* = Structure coefficients; *α* = Cronbach’s alpha.

**Table 3:** Descriptive statistics of the Portuguese version of the Intrinsic Religious Motivation Scale (IRMS) (10 items).

Variables	*M*	*SD*	*Min*	*Max*	*Min*	*Max*	*α*
Total IRMS	3.24	0.89	1.20	5.00	1.20	5.00	0.85
Intrinsic IRMS	3.26	1.17	1.00	5.00	1.00	5.00	0.92
Extrinsic IRMS	3.18	1.09	1.00	5.00	1.00	5.00	0.68

Note: *M =* Mean; *SD* = Standard deviation; *Min* = Minimum; *Max* = Maximum; *α* = Cronbach’s alpha.

**Table 4:** Exploratory Factor Analysis of the Centrality of Religiosity Scale (CRS), 5 items (*n* = 326).

Variables	*h^2^*	*L1*
1. How often do you think about religious issues?	0.71	0.84
2. To what extent do you believe that God or something divine exists?	0.77	0.88
3. How often do you take part in religious services?	0.57	0.76
4. How often do you pray?	0.70	0.84
5. How often do you experience situations in which you have the feeling that God or something divine intervenes in your life?	0.79	0.89
Eigenvalues		3.54
Total explained variance (%)		70.73
Correlational matrix range [0.30–0.90]	0.52–0.80	
Determining value [above 0.00001]		0.048
Bartlett’s Test of Sphericity (*df*); *p* < 0.05	977.45 (10); <0.001	
Kaiser-Meyer-Olkin (KMO) measure (above 0.50)		0.86
Diagonal element of the anti-correlational matrix (above 0.50)	0.82–0.90	
Cronbach’s alpha (*α*)		0.89

*Notes: h^2^* = Extracted communality coefficients; *LD* = Structure coefficients; *α* = Cronbach’s alpha.

**Table 5:** Descriptive statistics of the Self-Compassion Scale (SCS) and reliability (*n* = 326).

Instrument and Subscales	*M ± SD*	*Min*	*Max*	*α* *	*α* **	*α* ***
Total SCS	3.21 ± 0.63	1.38	5.00	0.92	0.89	0.91
Self-kindness	3.17 ± 0.85	1.00	5.00	0.78	0.84	0.86
Self-judgment	3.10 ± 0.90	1.00	5.00	0.77	0.82	0-84
Common humanity	3.35 ± 0.85	1.00	5.00	0.80	0.77	0.79
Isolation	3.20 ± 0.96	1.00	5.00	0.79	0.75	0.80
Mindfulness	3.35 ± 0.78	1.00	5.00	0.75	0.73	0.75
Over identification	3.14 ± 0.94	1.00	5.00	0.81	0.78	0.80

Note: *M* = Average; *SD* = Standard deviation; *Min* = Minimum; *Max* = Maximum; *α* = Cronbach’s alpha; * = values related to the study of Neff [36] ** = values related to the instrument validation study [54]; *** = values referring to this study.

**Table 6:** Correlations between the Intrinsic Religious Motivation Scale (IRMS) and the Self-Compassion Scale (SCS), Composite Reliability (CR), average variance extracted (AVE), and square roots of average variance extracted.

Variables	1	2	3	4	5	6	7	8	9	10	AVE	CR
1 Total IRMS	**0.814**	0.93 **	0.41 **	−0.02	−0.04	−0.04	0.07	−0.07	0.00	−0.00	0.662	0.951
2 Intrinsic IRMS		**0.827**	0.05	−0.03	−0.09	0.00	−0.00	−0.03	−0.05	0.03	0.684	0,937
3 Extrinsic IRMS			**0.781**	0.02	0.12 *	−0.10	0.19 **	−0.12 *	0.12 ^*^	−0.08	0.680	0.823
4 SCS Total				1	0.78 **	0.73 **	0.55 **	0.73 **	0.71 **	0.77 **		
5 SCS Self-kindness					1	0.38 **	0.60 **	0.30 **	0.74 **	0.34 **		
6 SCS Self-judgment						1	0.07	0.66 **	0.21 **	0.67 **		
7 SCS Common humanity							1	0.04	0.66 **	0.11 *		
8 SCS Isolation								1	0.25 **	0.78 **		
9 SCS Mindfulness									1	0.33 **		
10 SCS Over identification										1		

Notes: * *p* < 0.05; ** *p* < 0.001; bold = square root of AVE.

**Table 7:** Correlations between the Centrality of Religiosity Scale (CRS) and the Self-Compassion Scale (SCS), Composite Reliability (CR), average variance extracted (AVE), and the square root of AVE.

Variables	1	2	3	4	5	6	7	8	AVE	CR
1 Total CRS	**0.843**	0.111 *	0.095	0.108	−0.006	0.126 *	0.065	0.072	0.711	0.925
2 Total SCS		1	0.781 **	0.733 **	0.546 **	0.727 **	0.711 **	0.769 **		
3 SCS Self-kindness			1	0.383 **	0.601 **	0.302 **	0.736 **	0.343 **		
4 SCS Self-judgment				1	0.067	0.662 **	0.205 **	0.665 **		
5 SCS Common humanity					1	0.04	0.662 **	0.114 *		
6 SCS Isolation						1	0.247 **	0.776 **		
7 SCS Mindfulness							1	0.332 **		
8 SCS Over identification								1		

Notes: * *p* < 0.05; ** *p* < 0.001; bold = square root of AVE.

## Data Availability

As part of consenting to the study, survey respondents were assured that raw data would remain confidential and would not be shared.

## References

[1] Beckerson W.T., Durkheim E., Swain T.W. (1916). The Elementary Forms of the Religious Life. Ir. Church Q..

[2] Leite Â., Vidal D.G., Dinis M.A.P., Sousa H.F.P., Dias P. (2020). The Relevance of God to Religious Believers and Non-Believers. Religions.

[3] James D.W. (1982). The Varieties of Religious Experience: A Study in Human Nature.

[4] Cohen A.B. (2009). Many Forms of Culture. Am. Psychol..

[5] Inzlicht M., Tullett A.M., Good M. (2011). The need to believe: A neuroscience account of religion as a motivated process. Relig. Brain Behav..

[6] Arnett J.J. (2007). Emerging Adulthood: What Is It, and What Is It Good For?. Child Dev. Perspect..

[7] Ano G.G., Vasconcelles E.B. (2005). Religious coping and psychological adjustment to stress: A meta-analysis. J. Clin. Psychol..

[8] Vitell S.J., Bing M.N., Davison H.K., Ammeter A.P., Garner B.L., Novicevic M.M. (2009). Religiosity and moral identity: The mediating role of self-control. J. Bus. Ethics.

[9] De Freitas M.H. (2017). Psicologia religiosa: Psicologia da religião/espiritualidade, ou psicologia e religião/espiritualidade?. Rev. Pist. Prax..

[10] Hindriks P., Verkuyten M., Coenders M. (2014). Interminority Attitudes: The Roles of Ethnic and National Identification, Contact, and Multiculturalism. Soc. Psychol. Q..

[11] Carneiro A., Sousa H.F.P., Dinis M.A.P., Leite Â. (2021). Human values and religion: Evidence from the european social survey. Soc. Sci..

[12] Wulff D.H. (1997). Psychology of Religion: Classic and Contemporary.

[13] Freud S., Freud S. (1976). Obsessive Actions and Religious Practices. The Standard Edition of the Complete Psychological Works of Sigmund Freud (Trans. & Ed. James Strachey) Volume IX (1906–1908).

[14] Barrett J.L. (2004). Why Would Anyone Believe in God?.

[15] Barrett J.L., Bulbulia J., Sosis R., Al. E.H. (2007). Keeping science in cognitive science of religion: Needs of the field. The Evolution of Religion: Studies, Theories, and Critiques.

[16] Kelemen D., Rosset E. (2009). The Human Function Compunction: Teleological explanation in adults. Cognition.

[17] Uzarevic F., Coleman T.J. (2021). The psychology of nonbelievers. Curr. Opin. Psychol..

[18] Anczyk A., Grzymała-Moszcyńska H. (2021). The Psychology of Migration: Facing Cultural and Religious Diversity.

[19] Ghorbani N., Watson P.J., Khan Z.H. (2007). Theoretical, Empirical, and Potential Ideological Dimensions of Using Western Conceptualizations to Measure Muslim Religious Commitments. J. Muslim Ment. Health.

[20] Allport G.W., Ross J.M. (1967). Personal religious orientation and prejudice. J. Personal. Soc. Psychol..

[21] Abdel-Khalek A.M. (2017). The Construction and Validation of the Arabic Scale of Intrinsic Religiosity (ASIR). Psychol. Behav. Sci. Int. J..

[22] Eskelinen V., Pauha T., Kunst J., Räsänen A., Jasinskaja-Lahti I. (2021). Exploring religiosity and attitudes towards Christians and non-believers among recent Muslim refugees to Finland. Int. J. Intercult. Relat..

[23] Hafizi S., Koenig H.G., Khalifa D.A. (2015). Psychometric Properties of the Farsi Version of Hoge Intrinsic Religiosity Scale in Muslims: A Brief Report. Pastoral Psychol..

[24] Esperandio M.R.G., August H., Viacava J.J.C., Huber S., Fernandes M.L. (2019). Brazilian validation of centrality of religiosity scale (CRS-10BR AND CRS-5BR). Religions.

[25] Rożnowski B., Zarzycka B. (2020). Centrality of religiosity as a predictor of work orientation styles and work engagement: A moderating role of gender. Religions.

[26] Francis L.J., Wilcox C. (1996). Religion and gender orientation. Personal. Individ. Differ..

[27] Ackert M., Maglakelidze E., Badurashvili I., Huber S. (2020). Validation of the Short Forms of the Centrality of Religiosity Scale in Georgia. Religions.

[28] Hoge R. (1972). A Validated Intrinsic Religious Motivation Scale. J. Sci. Study Relig..

[29] Batson C.D. (1976). Religion as Prosocial: Agent or Double Agent?. J. Sci. Study Relig..

[30] Batson C.D., Schoenrade P.A. (1991). Measuring Religion as Quest: 1) Validity Concerns. J. Sci. Study Relig..

[31] Batson C.D., Schoenrade P.A. (1991). Measuring Religion as Quest: 2) Reliability Concerns. J. Sci. Study Relig..

[32] Huber S. (2003). Zentralität und Inhalt: Ein Neues Multidimensionales Messmodell der Religiosität.

[33] Huber S., Huber O.W. (2012). The Centrality of Religiosity Scale (CRS). Religions.

[34] Park C.L. (2007). Religiousness/spirituality and health: A meaning systems perspective. J. Behav. Med..

[35] Glock C.Y. (1962). On the study of religious commitment. Relig. Educ..

[36] Neff K.D. (2003). The Development and Validation of a Scale to Measure Self-Compassion. Self Identity.

[37] Krok D., Zarzycka B., Telka E. (2021). The Religious Meaning System and Resilience in Spouse Caregivers of Cancer Patients: A Moderated Mediation Model of Hope and Affect. J. Relig. Health.

[38] Appel J.E., Park C.L., Wortmann J.H., van Schie H.T. (2020). Meaning Violations, Religious/Spiritual Struggles, and Meaning in Life in the Face of Stressful Life Events. Int. J. Psychol. Relig..

[39] Nash D., Stark R., Glock C.Y. (1968). American Piety: The Nature of Religious Commitment. Soc. Forces.

[40] Seligman M.E., Csikszentmihalyi M. (2000). Positive psychology. An introduction. Am. Psychol..

[41] Forsman A.K., Wahlbeck K., Aarø L.E., Alonso J., Barry M.M., Brunn M., Cardoso G., Cattan M., De Girolamo G., Eberhard-Gran M. (2015). Research priorities for public mental health in Europe: Recommendations of the ROAMER project. Eur. J. Public Health.

[42] Kotera Y., Ting S.H. (2021). Positive Psychology of Malaysian University Students: Impacts of Engagement, Motivation, Self-Compassion, and Well-being on Mental Health. Int. J. Ment. Health Addict..

[43] Gilbert P. (2010). The Compassionate Mind: A New Approach to Life’s Challenges.

[44] Neff K.D., Kirkpatrick K.L., Rude S.S. (2007). Self-compassion and adaptive psychological functioning. J. Res. Personal..

[45] Yarnell L.M., Stafford R.E., Neff K.D., Reilly E.D., Knox M.C., Mullarkey M. (2015). Meta-Analysis of Gender Differences in Self-Compassion. Self Identity.

[46] Ghorbani N., Watson P.J., Kashanaki H., Chen Z.J. (2017). Diversity and Complexity of Religion and Spirituality in Iran: Relationships with Self-Compassion and Self-Forgiveness. Int. J. Psychol. Relig..

[47] Ghorbani N., Watson P.J., Chen Z., Norballa F. (2012). Self-Compassion in Iranian Muslims: Relationships with Integrative Self-Knowledge, Mental Health, and Religious Orientation. Int. J. Psychol. Relig..

[48] Rezapour-Mirsaleh Y., Abolhasani F., Amini R., Choobforoushzadeh A., Masoumi S., Shameli L. (2020). Effect of Self-Compassion Intervention based on a Religious Perspective on the Anxiety and Quality of Life of Infertile Women: A Quasi-Experimental Study. Research Square.

[49] De Souza L.K., Reppold C.T., Tavares I., Hutz C.S. (2020). Self-compassion in religious practitioners: Criterion validity evidence for the Self-Compassion Scale—Brazil. Psico.

[50] (2013). World Medical Association declaration of Helsinki: Ethical principles for medical research involving human subjects. JAMA J. Am. Med. Assoc..

[51] Allport G.W. (1950). The Individual and His Religion.

[52] Feagin J.R. (1964). Prejudice and Relegious Types: A Focused Study of Southern Fundamentalists. J. Sci. Study Relig..

[53] Raes F., Pommier E., Neff K.D., Van Gucht D. (2011). Construction and factorial validation of a short form of the Self-Compassion Scale. Clin. Psychol. Psychother..

[54] Castilho P., Pinto-Gouveia J., Duarte J. (2015). Evaluating the Multifactor Structure of the Long and Short Versions of the Self-Compassion Scale in a Clinical Sample. J. Clin. Psychol..

[55] Kaiser H.F. (1974). An index of factorial simplicity. Psychometrika.

[56] Bartlett M.S. (1950). Tests of Significance in Factor Analysis. Br. J. Stat. Psychol..

[57] Kaiser H.F. (1960). The Application of Electronic Computers to Factor Analysis. Educ. Psychol. Meas..

[58] Carpenter S. (2018). Ten Steps in Scale Development and Reporting: A Guide for Researchers. Commun. Methods Meas..

[59] Satorra A., Bentler P.M. (2001). A scaled difference chi-square test statistic for moment structure analysis. Psychometrika.

[60] Hu L.T., Bentler P.M. (1999). Cutoff criteria for fit indexes in covariance structure analysis: Conventional criteria versus new alternatives. Struct. Equ. Model..

[61] Browne M.W., Cudeck R. (1992). Alternative Ways of Assessing Model Fit. Sociol. Methods Res..

[62] Kline R.B. (2015). Principles and Practice of Structural Equation Modeling.

[63] Fornell C., Larcker D.F. (1981). Structural Equation Models with Unobservable Variables and Measurement Error: Algebra and Statistics. J. Mark. Res..

[64] Field A. (2009). Discovering Statistics Using SPSS.

